# The pressures of functional differentiation: education, homework, and schooled families

**DOI:** 10.3389/fsoc.2026.1848605

**Published:** 2026-08-26

**Authors:** Raf Vanderstraeten, Julio Labraña, Tomás Koch

**Affiliations:** 1 Department of Sociology, Ghent University, Ghent, Belgium; 2 Department of Education, Universidad de Tarapacá, Arica, Chile; 3 Faculty of Social Sciences, Universidad de Playa Ancha, Valparaíso, Chile

**Keywords:** history of homework, Niklas Luhmann, schooled society, structural coupling, system differentiation

## Abstract

The idea of functional differentiation is central to the framework which Luhmann developed for observing and analyzing the complexity of modern society. He analyzed its key social systems as functionally differentiated and specialized systems, such as the economy, politics, science, law, religion, or education. The modern family, however, is often defined as a multi-functional institution. It is seen to fulfill and integrate different functions, both for the adult members (emotional and psychological support, love) and for the children (education). Departing from Luhmann's framework, we examine whether and how such a multi-functional institution fits within a context defined by functional differentiation. We look at the structural pressures with which the family is confronted, especially in educational contexts. We argue that the school system has increasingly become able to claim jurisdiction over education. We look at specific forms of structural coupling, such as homework, which the school system uses to take control of its dependence on the family, and discuss why the family is now often reduced to some kind of preparatory institution, whose yardstick, as far as education is concerned, ultimately is their children's success at school. We conclude with more general reflections on the characteristics of functional specialization and differentiation.

## 1 Introduction

Niklas Luhmann's academic career spanned a period of about four decades. His first academic publications date from the late 1950s and the early 1960s (Luhmann, 1997). *Die Gesellschaft der Gesellschaft*, the last major publication which Luhmann saw into print, appeared in 1997, a few years after he had retired from the University of Bielefeld (Germany) and about 1 year before his death.

Luhmann was a highly prolific writer; his publication list includes nearly 2,500 items!^1^ Although he wrote about a very wide range of topics, his academic output is characterized by much coherence. From the early 1960s onwards, he focused on the articulation of a theory of society. He often also put emphasis on the consistency of this lifelong project. In the preface to *Die Gesellschaft der Gesellschaft*, for example, he proudly wrote:

“On my appointment to the Department of Sociology established at the University of Bielefeld in 1969, I was asked what research projects I had running. My project was, and ever since has been, the theory of society; term: thirty years; costs: none. As far as the time frame was concerned, my estimation of the difficulties was realistic” (2012, p. xi).

The idea of functional differentiation constitutes a, if not *the*, key element of his theory of modern society. Functional differentiation is distinguished from other forms of differentiation, particularly from segmentation, stratification and center-periphery differentiation. As Luhmann argued, modern society structures itself around functions to be fulfilled at the level of society . The transition to this form of differentiation means that

“the schematic of differentiation … [becomes] directed only by the functional problems of the societal system itself.” (Luhmann, 1995, p. 193)

In many publications, he analyzed the transition to functional differentiation and its consequences in the western world at the structural and the semantic level. Several voluminous monographs were devoted to analyses of single function systems, such as law, science, politics, the economy, art, religion, or education. There is little doubt that the idea of functional differentiation is at the core of the sociological legacy that Luhmann bequeathed us (e.g., Stichweh, in press; Baecker and Lehmann, in press). For Luhmann, the idea of functional differentiation is key to understanding modern society.

We respond to some of the ideas presented by the editors of this special issue on Luhmann. Building on Luhmann's work, as well as on work in which it has been critically discussed in the past decades, this paper intends to contribute to our understanding of functional differentiation. We particularly look at the relationship between the family and the school, and ask how this relationship has changed as a consequence of the expansion of schooling in the modern era. The family is a kind of outsider in the schematic of functional differentiation (see also Luhmann, 1990a, p. 196–217; Luhmann, 2017, p. 793–798). It is often seen to constitute a multi-functional institution, which, despite pressures for functional specialization, is still expected to combine several functions. Mostly reference is made to the relevance within/for the modern family of intimate relations (“love”) and of the care for and education of the children. At times, economic functions (joint consumption, joint investments) are also added to the list. Departing from Luhmann's framework, we examine in this paper how the modern family fits within a context defined by functional differentiation. We look at the structural pressures with which it is confronted, especially in educational contexts, where the “natural rights” of the family compete with the “professional claims” of the school.

Reflecting on the specifics of functional differentiation, this paper presents an analysis of historical changes in the dominant expectations regarding education in modern society. To be able to deal in some depth with the tensions and strains, generated by the form of functional differentiation, we more specifically include a case-study of how a particular form of coupling between the family and the school, viz. homework, emerged and became institutionalized in the western part of the world. As we argue, changes in the pedagogical views on homework are able to shed light on basic shifts in the structural coupling between the family and the school and in their “jurisdictional” authority over education. They allow us to analyze how the increased expectations of the school vis-à-vis the family have become normalized, and how in the case of education functional differentiation has historically taken shape. By combining this case-study with more general reflections on functional differentiation, we also aim to respond to questions and criticisms addressed in the past decades at Luhmann's work on functional differentiation (e.g., Tyrell, 1978, 2006; Berger, 2003; Schimank, 2005, 2015; Schwinn, 2011; Taylor, 2025), and to clarify the ways in which it can be used within sociological research today. The proof of the pudding indeed is in the eating.

In the following, we develop our argument in more detail. In the next section, we start with a brief presentation of some specifics of Luhmann's view on functional differentiation and its link with his theory of modern society. Afterwards, we briefly discuss the position of the school system in present-day society. In the fourth section, we look in more detail at the “invention” of homework, at how it increasingly gained legitimacy in the course of the last century, and at how it serves to couple the family and the school in ways which strengthen professional, school-based views of education. This case-study shows how families are being schooled, thus how the life of families with children has increasingly become oriented to specific standards and expectations defined by the school system. We conclude this article with a more general discussion of functional differentiation and the pressures exerted by this differentiation form on social systems.

## 2 Functional differentiation

An instructive way to clarify Luhmann's view on functional differentiation is to compare and contrast it with that of some of his predecessors, such as Georg Simmel, Emile Durkheim, and Talcott Parsons. We here focus on Parsons's view, as Luhmann explicitly worked “after Parsons” (both in the sense of: in the time after and in the spirit of) and modified this theoretical approach in fundamental respects (Vanderstraeten, 2022).^2^ Some of the connotations associated with “Parsonian” systems theory still determine how this approach is assessed today, despite the fact that Luhmann frequently explored directions that are completely different from Parsons's, both theoretically and historically.

As far as Parsons presented his work as systems theory, he put emphasis on the unity or “systemness” of social systems. In his reflections on functional differentiation, the idea of “system needs” played a central role. Parsons detailed the needs that had to be met if a social system were to survive. In his later work, this model was identified by the acronym AGIL, from the first initial of each of the four basic needs (see also Luhmann, 1988). He distinguished between the system needs of Adaptation (the problem of adapting to the environment and acquiring sufficient resources), Goal attainment (the problem of setting and implementing goals), Integration (the problem of maintaining solidarity or coordination among the subunits of the system), and Latency (the problem of creating, preserving, and transmitting the system's distinctive culture and values). According to Parsons, all social systems had to satisfy these four basic system needs. Complex systems, however, tend to differentiate along the lines of this frame; the functional imperatives (or “system needs”) generate the fault lines along which system differentiation occurs in complex societies (e.g., Parsons and Platt, 1973).

Parsons's analyses of functionally differentiated subsystems are determined by this view. In *Economy and Society*, which is the first of a series of publications devoted to the major differentiated societal subsystems, for example, the economy is defined as

“a special type of social system. It is a functional sub-system of the more inclusive society, differentiated from other sub-systems by specialization in the society's adaptive function.” (Parsons and Smelser, 1956, p. 306)

As part of the larger society, its primary function is the solution of the adaptive problems of society (as opposed to the problems related to the other “system needs”). And, as a differentiated subsystem of the larger system,

“the economy, like any social system, exchanges inputs and outputs over its boundaries with its situation. The most important part of the situation for the economy consists of the other cognate functional sub-systems of the same society and the institutionalized value system of the society.” (Parsons and Smelser, 1956, p. 307)

Put simply: Parsons put stress on the function of the parts for the whole and on the unity or “systemness” of the encompassing societal system (cf. Parsons, 1966, 1971).

A similar perspective is characteristic of Parsons's analyses of the modern family and of education. Writing in the mid-20th century, Parsons argued that the family had been able to adapt itself to the specialization and differentiation trends characteristic of modern society. Several subsystems (including the economy) had differentiated from the family, but, in Parsons's view, the family had not become an empty shell. Although it had lost some functions, the modern family had become able to intensify its investments in the remaining ones.

“The family is more specialized than before, but not in any general sense less important, because the society is dependent *more* exclusively on it for the performance of *certain* of its vital functions.” (Parsons and Bales, 1955, p. 10–11)^3^

The family was responsible for the biological reproduction of the human species and remained an economic unit (although mainly in terms of shared consumption, not of household production). More importantly, it could specialize in two other functions, which were increasingly “vital” for society:

“The effect of the process of differentiation is a sharp concentration on the two functional areas we have indicated as articulating personalities with the social system; namely, the socialization of children and the … [psychological stability of] adults.” (Parsons and Bales, 1955, p. 354)

The modern family was a multi-functional unit (and not a single-functional unit such as schools, factories, courthouses, etc.), but it fitted in a social order, dominated by functional differentiation. Moreover, as Parsons argued, the combination of these functional areas within a single institution (the modern, conjugal family) was likely to be of benefit at the level of the societal system (ibid.).

In terms of the socialization and education of children, the investments of the family had to be coordinated with those of schools. Using one of his “pattern variables,” Parsons argued that children learned or should learn their community's “particular” values in their family, while schools should acquaint them with society's “universalistic” values. He did not expect much conflict or divergence between these two subsystems, because they “have similar functions” (Parsons and Bales, 1955, p. 399).

Despite their differences, these subsystems are or should be well-integrated, as they orient themselves to the same general value system.

“Probably the most fundamental condition underlying this process [of socialization and education] is the sharing of common values by the two adult agencies involved – the family and the school.” (Parsons, 1959, p. 309)

In Parsons's view, functional differentiation could go along with frictions or strains within modern society. Parsons did not exclude the possibility of temporary tensions between distinct functionally specialized subsystems, such as the economy and the family. Although these subsystems are part of, and hence integrated in, the encompassing societal system, they also adhere to different rationales and have to give priority to different “system needs.” But in the case of the family and the school Parsons did not seem to expect structural frictions or strains. The “systemness” of the family and the school was in his view doubly controlled: these subsystems belonged to the same societal system *and* to the same subsystem, viz. education. They adhered (or had to adhere) to the same general *and* the same functionally specialized value system (see also Parsons, 2007, p. 334–385).

In this regard, Luhmann's (and our) perspective diverges fundamentally from Parsons's (see also Stichweh, 2013). Systems theory is *not* used to put emphasis on the “systemness” of social complexes. System differentiation is *not* perceived as the differentiation of the parts within an encompassing whole, but rather as the genesis and institutionalization of new boundaries, of new differences. And, in our view, Luhmann's emphasis on differences (instead of unity) or noise (instead of order) could also inform another perspective on the relationship between the family and the school.

Let us briefly clarify some theoretical differences. In an early, programmatic article, which sketches the outlines of his revised theory of functional differentiation, Luhmann started off by emphasizing the limitations of the traditional and Parsonian parts-whole framework and the promises of a new system-environment perspective:

“The most important contribution of [recent developments in] systems theory has been a change in the conceptual framework in terms of which systems are conceived and analyzed … [Systems theory] replaced the classical conceptual model of a whole that consists of parts and relations between parts by a model that focused on the difference between system and environment.” (Luhmann, 1977, p. 30)

My intention, he added,

“is to use the distinction of system and environment to work out a theory of system differentiation for the social system of the society” (ibid.).

The concept of system thus presupposes the concept of environment and vice versa. It directs attention to forms of closure, loose coupling and difference.^4^

A very similar argument is presented in Luhmann's late work. In the two-volume monograph *Theory of Society*, the last book publication which he saw into print, Luhmann (2012, p. 40) wrote that his intention has always been to understand society “as a system; and the form of the system … is nothing other than the distinction between system and environment.” In the chapter on the differentiation of society, he added:

“System differentiation does not mean that the whole is divided into parts and, seen on this level, then consist only of the parts and the ‘relations' between the parts. It is rather that every subsystem reconstructs the comprehensive system … through its own (subsystem-specific) difference between system and environment.” (2013, p. 3)

For Luhmann, systems theory has always been a tool to do away with the traditional connotations of the concept of system. He did not think of society as an organized or well-integrated social system, which possesses the qualities typically connected with “systemness” and normatively guaranteed unity. He rather called for careful historical analyses of the historical contingencies characteristic of the differentiation of society's function systems and the strains which ensued from these differentiation processes.

Along these lines, Luhmann also proposed to look at the differentiation of the education system. In his view, education had been “captured” by the dynamics of functional differentiation at a relatively late moment in history. Only after the differentiation of function systems for politics, economics, religion, and, in part, also for science, the prospects for education gradually began to change in the 18th century in Europe (and later in all other parts of the world as well). But he also expressed doubts about the new arrangement:

“As with the completion of a puzzle, the pieces that have already been differentiated (from the others) have a suggestive influence on what can possibly and must necessarily be connected to them. But, unlike with a puzzle, it is not certain from the outset that a complete picture will be produced or that it will be understandable as a whole.” (Luhmann and Schorr, 1988, p. 24)

The same doubts are expressed in several other publications (e.g., Luhmann, 2026). Throughout his scholarly work, Luhmann always remained skeptical about how, intentionally or unintentionally, overtly or latently, the differentiated education system prepared individual human beings for participation in modern society (Vanderstraeten, 2000).

The differentiation of function systems, as Luhmann argued, is not just controlled by encompassing system needs or ideals of “systemness” and wholeness. Function systems, such as education, have to adapt themselves to other function systems in their social environment, and vice versa: the social environment has to adapt to the differentiating function systems.^5^ In general terms, the question then is how differentiated function systems fit with other pieces of the puzzle of modern society. In more specific terms, the question is what kind of “structural coupling” has developed between these systems, what kind of “selective filter” canalizes the influences on and between them. Without direct control, such couplings define the ways in which the different parts fit together.

Such kind of analysis of the education system has not been systematically explored by Luhmann, although one can find some scattered remarks in his many publications on education. Building on Luhmann's perspective on system differentiation, we argue that it is useful to look in more detail at the relationship between the family and the school. Quite different from Parsons's perspective, with its focus on systemic partnership and system integration, we expect to find evidence of historical tensions between the family and the school (see also Tyrell, 1985, 1987; Tyrell and Vanderstraeten, 2017; Vanderstraeten, 2023). We also argue that Luhmann's perspective on system differentiation is able to provide us with the tools necessary to make sense of these structural tensions. Divergences and differences between the systems of the family and the school are, in our view, more than temporary problems that need to be solved. They are indices of basic structural characteristics of this relationship. They can also be studied to shed light on historical shifts in the relationship between the family and the school. We argue, in addition, that the rapid expansion of schooling has changed how both systems are structurally coupled, and that the societal prestige of schooling reduces the legitimacy of the contributions which families can make to education.

## 3 Greedy institutions

Various indicators can be used to document and analyze the expansion of schooling (see Kavadias et al., 2025). As we will see, a reflection on the growing visibility and growing impact of formal schooling in the present-day “schooled society” (Baker, 2014) is helpful to make sense of the differentiation of the education system and of changes in the structural coupling between the family and the school. Such a reflection displays how the structural coupling between the family and the school currently mostly forces the family to adapt to the claims to expertise of the school.

In line with many contributions written from the perspective of the so-called new institutionalism, the expansion of schooling is now often characterized as a modern, global phenomenon. However, it should not be forgotten that school participation has long been low or limited (from our present-day perspective). In 1961, for example, more than two-thirds of the total adult population of a typical modern country such as Belgium had only completed primary education and had left school at the age of 14, which was at that time the age at which mandatory schooling ended (Vanderstraeten and Van der Gucht, 2023). In many other parts of the world, too, the majority of each new generation was long not included after the compulsory school age. In the past decades, however, school participation has expanded rapidly. Enrolment in primary and secondary education has become compulsory in many places, while enrolments in postsecondary institutions have also grown apace. The proportion of an age cohort going on from secondary school graduation to higher education is nowadays often more than 50 per cent—a situation historically unprecedented (Baker, 2014).

A clear indication of the changing expectations with regard to school participation is the fact that the distinction between high- and low-schooled individuals has acquired special importance in recent decades. In a period in which it becomes more or less self-evident to participate in tertiary education, it also becomes problematic *not* to finish school and graduate. Early school leaving (or dropout) is presently perceived as a form of failure, just as university graduation equalizes success. In this sense, the “school dropout” is the opposite of the university graduate. But it should also be emphasized that the “school dropout” became perceived and dealt with as a “social problem” in a period in which the school system was rapidly expanding and in which the number of people dropping out of school was declining sharply. It did not exist before the rapid expansion of secondary and higher schooling in the last decades. It is thus in a specific sense a “socio-historical” problem; it is an identification of deviant behavior, of not living up to the changing social expectations regarding school participation. It is a consequence of the growing pressure to finish school or university and graduate (Dorn, 1996; Baker, 2014; Vanderstraeten and Van der Gucht, 2023; see also Vanderstraeten, 2015).

Since the last decades of the 20th century, Baker (2014) has argued, the school system no longer just fits within, or is controlled by, existing societal structures, but asserts itself wide-ranging relevance in society. This means, in Baker's view, that schooling has transformed from a secondary institution into a primary, foundational authority that is shaping the broader cultural level and the currently dominant ideas about the “good society.” Baker speaks of “the schooled society,” because, in his view, schooling has come to influence about “everything” from economic development and political systems to individual cognitive development and social stratification (Baker, 2014, 2025; see also Frank and Meyer, 2020; Kavadias et al., 2025). Despite the merits of Baker's analysis, such a generalization probably goes too far. Following Luhmann's idea of functional differentiation, it is not difficult to see that many function systems have been able to leave their mark on society. And many characterizations of society have been highlighting the (un-)importance of particular function systems, including “secular society” (religion), “capitalist society” (economy), “democratic society” (politics), or “knowledge society” (science). Following the rapid expansion of schooling, it is possible to add schooling to the list of influential systems, but the length of this list itself should also remind us of the need to relativize the impact of any one single function system on society as a whole. The pattern of functional differentiation allows in principle many function systems to hypostatize their own *raison d'être*. Specific historical conditions may of course determine the range of available options. But, although the expansion of the school system may be seen to lead to the “educationalization” and “pedagogicalization” of society, the schematic of functional differentiation goes along with loose coupling and heterogeneity within society. And the ensuing diversity and heterogeneity at the level of the contemporary societal system may be seen to stand in opposition to the restrictions that are likely to go along with the dominance or monopoly of one single function system.

There can be little doubt, however, that schooling is now widely seen to be a “just” and legitimate source of social authority. School credentials are generally held to be preferable to other credentials (such as family-based economic or social “capital”). There is much evidence from international value studies (i.e., studies which focus on what adults believe and value about education) that most adults agree that schooling is *the* legitimate way to prepare for life, and that educational achievement is generally a fair and uncontroversial way to determine responsibilities and commensurate rewards in adulthood. And, not surprisingly, adults with higher credentials and thus with more exposure to the school system also hold the strongest beliefs in its societal authority. For the new institutionalism, schooling has become strongly institutionalized within contemporary society. The differentiated and expanding system of schooling, we might add, is increasingly able to assert itself in society.

If we look at the consequences of the expansion of school participation from a historical perspective, it makes sense to characterize schools as “greedy institutions,” because, in the words of Coser (1974, p. 4),^6^ they “seek exclusive and undivided loyalty and … attempt to reduce the claims of competing roles and status positions on those they wish to encompass within their boundaries.” Coser did not explore this perspective on schools himself; he focused on the members of institutions such as religious sects, utopian communities and ideal-typical American middle-class families (especially housewives and domestic servants). But, following authors who emphasize the prominent position of schools within contemporary society (see Meyer and Rowan, 1983; Baker, 2014, esp. p. 279–282), it can be argued that schools are now in a position to significantly interfere with their students' roles or involvements in other social institutions. The demands which schools make often encroach upon various other aspects of their students' life, including the life of other family members and of the family as a whole. Schools now seem able to exercise much pressure on the family, thereby reducing the family to some kind of preparatory function, whose yardstick, as far as education is concerned, ultimately is their children's success at school (see Luhmann, 2002, p. 111).

From this perspective, we explore in the next section in more historical detail how the school system has been able to export its standards to its social environment, especially to families. For sure, the boundaries between these institutions have at times become blurred—as in the case of boarding schools, where children live in “family-like” settings on the premises of the school during large parts of the year, or as in the case of homeschooling, which is the education of “school-aged” children at home, which has at several places in the world become a legal alternative to public schooling. But, despite such “mixed” social institutions, most children grow up in families *and* schools. Focusing on homework, we particularly analyze how the school system aims at ensuring its own “systemness” by adapting the socially relevant environment of the family to its “system needs” (Luhmann, 2004, p. 123–158)^7^.

As we will see, the ways in which the “output” of the school system is being valued within its societal environment (and especially within the labor market) puts pressure on the relationship between the school and the family; it backs up the initiatives and interventions of the school system that aim at reducing the impact of family *differences* on the school achievements of children and at strengthening the ways in which families provide support to the demands of schools. The “value” of the family is increasingly measured in terms of the children's school success.

## 4 Pressures of functional differentiation

How functional differentiation takes effect, Luhmann argued, is best analyzed via the distinction between system and environment. Departing from this distinction, attention needs to be directed to the autonomy (or “autopoiesis”) and the boundary-work of social systems. Differentiated systems close themselves off from their environment, but Luhmann's notion of autonomy does not imply that these systems are independent from their environment (e.g., Luhmann, 1986, 1995, p. 176–209). This notion rather implies that these systems search for ways to take control of the relevant “input” from their environment. Differentiated systems, so Luhmann (1977, p. 36), “can afford indifference against anything except very special traits of their respective environment.” They use forms of structural coupling to canalize the influences of their environment on them.

For differentiated systems, many differences may thus not make a real difference, to adapt Gregory Bateson's famous phrase. But some differences in the environment do make a difference in the system. In this light, we might ask how systems perceive their environments, and how they respond to or act upon what they consider to be relevant factors in their environments (see, e.g., Luhmann, 2013, 2017). For our case-study, the question is how the relationship between the family and the school has taken shape socio-historically, and what the characteristics of the relationship between these two systems/environments tell us about the differentiation of education.

That the family and the school are relevant to one another was itself for a long period of time not self-evident. In the early-modern era, the family and the school were for the most part perceived as alternatives or opposites. Typically, “good” parents were not the ones who sent their children to a school, but the ones who did not. Schools were not defined in relationship to family households. They were seen as a symptom of the family's failure; to send a child to a school was widely thought to reflect unfavorably on the family (see Tyrell and Vanderstraeten, 2017; Vanderstraeten, 2023). The boarding schools, which were expanding at that time, also seemed able to completely replace the family. Echoes of such perceived incongruity between both institutions can still be found in the 18th and early-19th-century reflections on education. Responding to the gradual expansion of formal schooling, it was, for example, doubted whether teachers could exercise the kind of “natural” authority that was thought to be required for education, when there existed no blood-ties between them and their pupils. The question of “natural” trust was also raised, especially because the mistakes of schoolteachers were seen to stand out more and be less easily excused than those of parents (e.g., Ehlers, 1766). But, in connection with the focus on knowledge of the European Enlightenment, the professional training and expertise of schoolteachers could also be used to question the “natural” primacy of the family in matters of socialization and education (e.g., Trapp, 1780; Condorcet, 1792; see also Lortie, 1975; Luhmann and Schorr, 1988, p. 79). Ways to redefine and restructure the relationship between the family and the school then also became visible.

Following the expansion of schooling, the school system gradually took the lead in restructuring this relationship. This system has become able to assert its “definition of the situation” in a variety of settings. The “invention” and the history of homework illustrate what kind of shift has taken place (although, as already mentioned, other cases can be explored as well to analyze these changes).^8^ In the mid-19th century, western school reformers, such as Horace Mann and Edward Thring, discussed homework as a new pedagogical practice. In Mann's *Lectures and Annual Reports on Education*, for example, homework was presented as a means of moral cultivation. Mann argued that study beyond school hours trained the will, built self-discipline, and allowed parents to observe the moral tenor of their children's private thoughts. Homework assignments had to stimulate children's moral growth; such an assignment “prepares the means, beforehand, of determining what those [moral] meditations shall be” (Mann, 1867, p. 64).

Although the home was the proper space for moral reflection and pedagogical reinforcement, this space had to be prepared and cultivated, and homework could extend the teacher's moral authority into the domestic sphere.

In Britain, Edward Thring advanced a markedly institutional conception of education that limited the role of families in defining school matters. But he concluded his treatise, titled *Education and School* (1867), with a chapter on the role of families:

“a treatise on schools would be imperfect which did not say something about homes. It is a delicate and dangerous subject to touch on; but after all, the homes first make the schools, before the schools have chance of making the homes.” (1867, p. 272)

Overall, he argued that questions of pedagogy and discipline belonged to professional educators. Families lacked the knowledge and perspective required to judge the internal operations of schools. The problem, he argued, was most serious when families did not have any clear understanding of educational principles and questioned “things which are absolutely in the power of schools to do, or not to do” (1867, p. 274).

Thring positioned the family as a potential source of interference, which at times seemed to unnecessarily question the regulating authority of the school. Families were invited to participate in education, yet only within the framework defined by expert teachers and schools. And homework was considered necessary to communicate these expectations.

By the late-19th century, some school reformers had begun to work more methodically on the extension of “the spirit of the school” (as Max Weber might have argued) into domestic life (see also Labraña et al., 2025; Vanderstraeten and Weinbach, 2026). Several contributors to the so-called Herbartian movement, which was characterized by a strong focus on instruction technology and lesson design (didactics), explicitly integrated homework into the didactic sequence. Within their model, the application phase required pupils to transfer and use classroom knowledge outside the walls of the school. Homework was situated in the stage of consolidation, the moral and cognitive fixation of what had been learned (e.g., De Garmo, 1899). The family home was thereby re-imagined as a kind of auxiliary classroom, where the lesson content, first presented by professional schoolteachers, had to be internalized through repetition and application.

However, these formalized models of instruction and learning soon also triggered criticism, especially from authors linked to the so-called progressive movement. At the turn of the 20th century, John Dewey, for example, questioned the value of repetitive assignments detached from experience. In *The School and Society*, originally published in 1899, Dewey (1915, p. 52) warned that homework fragmented learning by separating the cooperative life of the school from the child's world at home. In his view, no distinction had to exist between “the ideal home” and “the ideal school.” Genuine education united knowledge and action; mechanical repetition conducted in isolation weakened both creativity and democratic participation. Montessori (1912/1964), who developed a child-centered educational approach that acquired much acclaim, argued for her part that schoolwork had to be done at school, not at home. She rejected homework entirely, insisting that classrooms with their own hands-on learning materials and “prepared environments” should suffice for intellectual and emotional growth. Work assigned by the school for the home reflected, in Montessori's view, a pedagogical failure to engage children meaningfully at school. Célestin Freinet, another well-known and influential reformer, shared this criticism, portraying homework as the antipode of self-directed learning and the symbol of authoritarian schooling and rigid teaching methods. He focused instead on cooperative projects rooted in community life, arguing that authentic learning should take place in the community rather than remain confined to the private sphere of the household (Freinet, 1964). Indeed, progressive educators long broadly condemned homework as a remnant of rote learning that suppressed curiosity and novel interests, exhausted families, and deprived them of valuable “family time” (see Gill and Schlossman, 1996, 2003; Cooper, 2007).

The “politics of homework,” as Gill and Schlossman (1996, 2003) have argued, whilst focusing on the 20th-century USA, constituted a recurring source of controversy. Early in that century,

“the vast majority of educational commentary on homework attacked its central place in schooling.” (Gill and Schlossman, 2003, p. 846)

By its opponents, it was viewed as a “sin against childhood” and a direct threat to the parents' authority to manage their children's activities outside of the school (Gill and Schlossman, 1996). By its proponents, on the other hand, it was perceived as a link that kept the parents informed about what the school was teaching and that gave them a chance to participate in their children's schooling. In this view, not to assign homework was to exclude the parents from playing an active role in their children's overall educational development (ibid.). But homework made it possible to “upgrade” the educational impact of the family.

Early in the 20th century, the

“disagreements about homework were far more heated than they would become later in the century.” (Gill and Schlossman, 2003, p. 848)

In the second half of the 20th century, homework became part of the educational mission of the “good school.” Next to educational arguments, broader world-political and world-economic tensions played their part. In the western part of the world, homework became entangled with anxieties about national strength and economic competitiveness. Whereas early-twentieth-century progressive educators had warned that excessive homework assignments harmed families and stifled creativity, postwar policymakers feared that too little homework signaled intellectual decline. Homework turned into a patriotic obligation, a moral counterpart to technological rivalry. At the end of the 20th century, such rhetorical strategies became commonplace (see also Cooper, 2007). Concerns about the broader dangers for national projects of “insufficient” homework have persisted to the present day, as a variety of official policy reports, from *A Nation at Risk* (1983) to the more recent PISA studies, illustrate.

In the latter part of the 20th century, prevailing ideas of the “good home” have been adapted to the expectations of schools and schoolteachers. Whatever their socio-economic or class background, parents have become expected to set up a homework-friendly area in their home, schedule a regular study-time for their children, keep distractions to a minimum, motivate their children, monitor their homework, and so on. Homework assignments transform the place called home into a setting where school norms are to be internalized and enacted (cf. Goodlad, 1984). Parents are also blamed, when they are perceived to pay insufficient attention to their children's homework and the demands of schools and teachers (see MacLure and Walker, 2000; Schaub, 2023; Vanderstraeten, 2023). More generally, the “good home” is now defined as the demanding home, where material conditions and parental attitudes are favorable to the children's achievements at school. The nearly daily ritual of homework assignments structurally anchors the logic of the school in the family.^9^

For sure, critical views on homework have not disappeared. For example, Popkewitz (2003) referred to the emergence of parents as trained, “pedagogicalized” educators, for whom parenting has become a deliberate pedagogical project, oriented to their children's learning outcomes. Parents, he argued (following Michel Foucault), are urged to monitor, motivate, and explicitly evaluate their children, turning parental love or affection into variables determined by their children's “normalized” performances within school contexts (see also Bruin and Nevøy, 2022; Colla, 2022).

From a gender perspective, the “moral economy” of homework has also been criticized. The ideal of the “good parent” is seen to be both moralized and gendered, positioning mothers at the core of educational surveillance and affective labor. Even when mothers question the pedagogical value of homework, homework is difficult to negotiate and many mothers continue to sustain its routines to avoid social disapproval. As, for example, Lehner-Mear (2021, p. 297) observes, “whilst homework might make children unhappy, its assumed educational value, combined with the good-mother-as-educator narrative, requires compliance.” According to Schaub (2023, p. 119), “the strong cultural belief” in schooling has led to new, invasive norms regarding “educationally strategic parenting.” This belief creates, as she writes, “new responsibilities for parents such as monitoring school performance, supervising homework, scheduling extracurricular activities, and acting as a liaison with education officials” (ibid.).

Probably only few parents are able to consistently meet the expectations imposed by schools, but the strong cultural belief in the authority of the school system makes it almost impossible to question the legitimacy of these expectations. Finding ways to adapt to these expectations has become a defining marker of responsible parenthood. This so-called “new partnership” between the family and the school thus extends the jurisdiction of the school in matters of education. It “normalizes” the ways in which the school system defines education.

We stop our short discussion of the “politics of homework” here. Many justifications have been and will continue to be used—both for and against homework. It is, in our view, unlikely that “objective” arguments can end the controversies. The “politics of homework” is the expression of institutional conflicts, or, as Abbott (2014) would say, of “jurisdictional conflicts” about the authority over education between the school system and the family. Homework materializes the structural coupling between the school and the family; it now serves to routinely extend specific expectations of the school in the intimate sphere of the family. It is both a factor in, and a catalyst for, the asymmetrical relationship that has become institutionalized between the family and the school.

To sum up: our analyses show how the dominant paradigm of schooling puts much pressure on the family. The school system has been able to take control of its dependence on the family, to impose various structural constraints on the family. A range of interventions has been informed by its views on how to optimally manage the relationship with the family. Practices, such as homework, are best interpreted as ways to control its dependence on the family, and to adapt the family to increased expectations of the school system. In the recent past, the jurisdictional authority over education of the school system has expanded rapidly. The schematic of functional differentiation also made it difficult for the “multi-functional” institution of the family to uphold its traditional jurisdictional claims and oppose the demands of the school system. The expanding jurisdictional authority of the school system has rather forced families to redefine how they observe and “realize” themselves.^10^ As a result, the structural coupling between the school and the family is highly asymmetrical.

The transition to functional differentiation made it possible and necessary to “reinvent” education. Following the diffusion of mass schooling, the school system could increasingly organize itself in its own terms. It could upgrade its self-referential autonomy: it became self-evident that schools use school tests to determine whether children can be admitted, and that school diplomas provide admission to the next level of the system—where students can pass more tests, pick up course credits, and earn other school diplomas. Building upon its organizational infrastructure, the school system could thus claim operational autonomy, and routinely take control of the distinctions relevant in the system (Vanderstraeten, 2002).^11^ As part of its boundary-work, it also tries to ensure control over the relevant ties with the environment. As families provide much input that is relevant to the learning processes that take place in school, forms of structural coupling have emerged that aim at controlling this dependency. Homework is one such example. These interventions have paved the way for later developments, which have further changed the ways in which the family and the school take part in education. Increased expectations vis-à-vis the family have become institutionalized. As we have seen, the educational input of the family has not become irrelevant to the school system, but the school system increasingly aims at taking full control over education, whereas the “multi-functional” institution of the modern family has difficulty opposing the jurisdictional claims of the school system.

## 5 Conclusion

Luhmann always emphasized the continuity (if not linearity) of his academic work. Early work was at times published under the title of “introductory” or “preliminary remarks,” while books such as *Die Gesellschaft der Gesellschaft* were explicitly presented as the culmination of his consistent, career-long work on the renewal of sociology's theory of society. At the same time, it is obvious that Luhmann, if he had lived longer, would have continued to revise and rewrite his theory of society. His “system” was always on the move (see also Stichweh, 2011).^12^ And, as more documents from the *Nachlass* are becoming publicly available in the last years, it is also surprising to see which (and how many) manuscripts and ideas Luhmann decided *not* to publish during his lifetime.

In part, the mobility of his “system” might explain why it is so difficult to summarize the different parts of Luhmann's theory of society, for example, for handbooks to be used in theory courses in sociology programs. The work of some of his contemporaries who had similar ambitions, such as Pierre Bourdieu, has much more readily become included in a “handy” format in the sociological canon. Although some “companions” to Niklas Luhmann now exist, and more are to be expected in the coming years, especially in relation with the celebration of the centennial of his birth, Luhmann remains a kind of enigmatic outsider, whose “system” of concepts and approaches is not often used as a toolbox for systematic research and systematic sociological reflection. Mirroring the reception of Parsons's work since the latter part of the 20th century, the highly abstract level of theorizing of Luhmann's “grand theory” and the difficulties of unambiguously specifying and applying its basic concepts are often decried, especially within the Anglo-Saxon sociological literature (for a recent example, see Taylor, 2025). Luhmann, just as Parsons several decades ago, is habitually depicted as an out-of-this-world theorist, whose work is largely void of empirical or historical relevance.

Against this background, we have in this paper presented both a reflection on some general sociological ideas and a case-study of the relationship between the family and the school. Taking up the challenges of a case-study is a way to test the usefulness of Luhmann's toolbox, while it can also be a way to stimulate a reflection on the “system” of concepts and approaches he left behind. As we have tried to illustrate, while focusing on functional differentiation, Luhmann not only created finished products, but also began things and opened up a broad range of perspectives that are worth further pursuing. Perhaps the primary significance of his work today is that it provides an important impetus for theory-driven and theory-directed sociological research.^13^

As other forms of differentiation, functional differentiation is for Luhmann a way of “dividing” society. Luhmann argued that the schematic of functional differentiation has become dominant or achieved “primacy” in society (e.g., Luhmann, 2013, p. 12). But differentiation processes are also historically contingent processes. Function systems, such as law, politics, the economy, science, art, religion or education, are marked by the historical conditions of their formation and by the opportunities and limitations that have emerged afterwards (see also Roth and Schutz, 2015; Vanderstraeten, 2022). Functional differentiation cannot be steered top-down; Luhmann time and again emphasized that this schematic leads to horizontal or “heterarchical” relations between function systems and their environments (e.g., Luhmann, 1982, 2013, p. 65–127). This view on differentiation might also be seen to underwrite the moral importance of diversity and singularity.

In short: the “systemness” of society is not a *fait accompli*. The relationships with (systems in) their environments rather produce a variety of challenges and problems in the differentiated function systems. Against this background, we have argued that the schematic of functional differentiation comes with pressures and tensions. While this schematic reinforces the legitimacy of certain views, it inhibits others. It provides support in certain directions, but also elicits conflicts and contradictions. For sociological analyses, Luhmann's view on functional differentiation is apt to foreground this historicized perspective on the relationship and the structural coupling between systems and their environments (e.g., Labraña and Billi, 2025; Labraña, 2026).

In most parts of the world, education was “captured” by the dynamics of functional differentiation at a relatively late moment in time. Families have long been able to claim authority over the education of their children. But the place of schooling in society has shifted in the course of the past century. Backed up by the schematic of functional differentiation, schools have become increasingly able to claim control over the ideas and ideals of “good education.” The structural couplings, which emerged between schools and other institutions within society, especially the labor market (often analyzed in terms of human capital investments), are providing support for a range of interventions in the family. The family is a relevant environment of the school, but it is being schooled. As a multi-functional institution, the family also has difficulty opposing itself to the pressures of functional differentiation. As we have seen in somewhat more detail, ideas and debates about homework, as presented in the past two centuries or so in the western part of the world, illustrate changes in the “jurisdictional authority” over education. They illustrate how structural couplings selectively translate specific educational claims of the school system into interventions in the family. They show how the increased expectations of the school vis-à-vis the “educational responsibilities” of the family have become normalized. Our case-study allows us to hypothesize that similar structural pressures are likely to characterize the evolution of other function systems as well.

## Data Availability

The original contributions presented in the study are included in the article/supplementary material, further inquiries can be directed to the corresponding authors.

## Footnotes

1 For this publication list, compiled by Klaus Dammann and the administrators of the *Nachlass*, see https://niklas-luhmann-archiv.de/person/nl-bibliographie. To be fair: this list includes all reprints and translations. Moreover, quite some unpublished (and at times incomplete) manuscripts from the *Nachlass* have been sent into print in the course of the past decade or so. But, as there are still other unpublished manuscripts, Luhmann's publication list is in the next years likely to become longer yet …

2 As Parsons always explicitly tried to build on authors, such as Durkheim, and thereby stand on the shoulders of giants (in the idiom of the day, which he preferred), we will also give attention to the history of the idea of functional differentiation within sociology. Durkheim's distinction between “mechanic solidarity” and “organic solidarity” is one between two types of differentiation, viz. segmentation and functional differentiation. Like Durkheim, Parsons was very much focused on the stability of the pattern of functional differentiation. As we will see, Luhmann presented a very different view on the stability and “systemness” of this order.

3 Without doubt, Parsons here responded to Emile Durkheim's view, presented in the introduction to the second edition of *De la Division du Travail Social*, that “the corporation” or “the professional group” had replaced the family as basic moral unit in modern society. The family had played “so important a role in the moral history of humanity,” but “by losing its former unity and indivisibility, [it] has lost at the same time much of its effectiveness” (Durkheim, 2013, p. 19). While Parsons did not question that the family had lost some of its former effectiveness, he argued that it had been able to reinvent itself as a support system for all its members. The modern family, characterized by the fact that adults are free to build a new family with a partner of their choice, continued to fulfil important functions in modern society in Parsons's view.

4 Let us add that Luhmann distinguished between two types of social differentiation: next to functional differentiation, he also elaborated on the differentiation between the levels of interaction, organization, and society. Discussions of the second type of differentiation were quite prominent in his early work, but became somewhat marginalized in his later work (for an exception, see Luhmann, 1990b). From the 1970s onwards, the distinction between system and environment was by Luhmann for the most part used to explore and analyze the complexities of the functional differentiation of modern society (see Tyrell, 2006).

5 Differentiating systems may thus affect systems in their environment in unpredictable and uncontrollable ways. To give an example: the introduction of compulsory school attendance (often in combination with the prohibition of child labor) cannot be reduced to a mere response to new “system needs” at the level of modern society, but rather constitutes a different environmental challenge for the economy, the political system, the health care system, the families, and so on. Each of these systems needs to react in its own ways.

6 Lewis Coser, who introduced the term, contrasted his “greedy institutions” with Erving Goffman's “total institutions,” which rely on physical barriers to separate inmates from the outside world. Coser's greedy institutions rely on voluntary compliance and do not physically separate their members from others, but they exert much pressure on members in situations that are not directly controlled by them.

7 Other case-studies might be explored as well. Another example could be the elaboration of truancy politics. National policies with regard to truancy differ, but almost everywhere schools are now able to enlist legal and political support for their jurisdictional claims. If pupils are absent from school for a long period of time, their parents may be fined. Local child services or social services officers may even petition a court to remove child custody from the parents. Hitherto most studies on truancy politics focus on the (in-)effectiveness of such interventions. But it is also useful to look into the relationship between such politics and the ways we think of the importance of schooling and what constitutes “appropriate parenting.”

8 Our case-study also focuses on debates in the western part of the world. It would, of course, be worthwhile to add analyses of other educational traditions, such as those in Asia, and the way they deal with the relationship between family and school settings. Although some instructive comparisons between educational traditions in different parts of the world have seen the light of the day in the past decades (e.g., Alexander, 2000), not much studies have hitherto systematically explored the specificities of the different educational traditions (see also Phuong and Vanderstraeten, 2024). Instead of focusing on the socio-historical embeddedness of educational traditions, much of the recent comparative educational literature has been preoccupied with the results and the country rankings of international student assessments, such as PISA or TIMMS. But this also often implies that the specific arrangements in the countries of which students score high on the tests are presented as the “best practices,” not as the outcome of historical evolutions and conflicts (see also Stichweh, 2025).

9 Importantly, empirical evidence in support of the main (also classical functionalist) argument, namely that homework enhances children's achievements at school, remains inconclusive. For example, the psychologists Trautwein and Köller (2003, p. 115) speak of “a mystery”: “Despite the long history of homework and homework research, the role that homework plays in enhancing student achievement is, at best, only partly understood.” And they conclude their research assessment as follows: “our review indicates that the discipline is still far from achieving one of its central goals—clearly articulating the strength of the relationship between homework and [school] achievement” (2003, p. 141). In other words, the debate about homework is a matter of defining and redefining the jurisdiction and authority over education.

10 We here adapt an expression of Pierre Bourdieu, according to whom the family is a “realized category.” According to Bourdieu (1996), “the dominant, legitimate definition of the normal family … is based on a constellation of words—house, home, household, *maison, maisonnée*—which, while seeming to describe social reality, in fact construct it” (1996, p. 19). In Bourdieu's view, the dominant vocabulary masks the underlying construction process, presenting the domestic unit as a “natural” phenomenon. But, as we argue, part of the definition of the normal family has come to include an orientation to the school achievements of children. The “good home” is now expected to give priority to the expectations of the school system.

11 We might here situate the origins of what John W. Meyer and Brian Rowan once called the “Schooling Rule.” In their words: “Education is a certified teacher teaching a standardized curricular topic to a registered student in an accredited school” (Meyer and Rowan, 1983, p. 73). Education is now imagined as a system of schooling. The school system provides an organizational platform for education, which could increasingly claim authority over education and close itself off from its environment, including the input of the many different families, where the school-going children are being socialized and educated in many different ways.

12 In an interview, taken in 1985, thus shortly after the publication of *Soziale Systeme*, Luhmann described everything he had published until that time as zero series production (“Null-Serie der Theorieproduktion”), adding that *Soziale Systeme* could constitute an exception. The follow-up question of the interviewer “Is this your first real publication?” is answered as follows: “Yes, it's certainly the starting point for the *Nachlass*, so to speak [i.e., what is going to follow]. I won't discard this book so quickly, even though I would already do some things differently” (Luhmann, 1987, p. 142, our translation).

13 These sentences are close to formulations that appear in an obituary, which Luhmann wrote for Parsons. “Talcott Parsons left his lifework unfinished. The form in which we find it is the result of a continuous theoretical development that lasted for decades … [But] a fair critique of his work would take a discouragingly great effort. Thus, there exists the danger that his work will be mummified and stored for later rediscovery, without proving fruitful to contemporary theoretical developments” (Luhmann, 1982, p. 47).
